# High reactivity of H_2_O vapor on GaN surfaces

**DOI:** 10.1080/14686996.2022.2052180

**Published:** 2022-04-08

**Authors:** Masatomo Sumiya, Masato Sumita, Yasutaka Tsuda, Tetsuya Sakamoto, Liwen Sang, Yoshitomo Harada, Akitaka Yoshigoe

**Affiliations:** a Next Generation Semiconductor Group, National Institute for Materials Science, Tsukuba, Japan; b Center for Advanced Intelligence Project (AIP), RIKEN, Tokyo, Japan; c Materials Sciences Research Center, Japan Atomic Energy Agency, Hyogo, Japan; d International Center for Materials Nanoarchitectonics (WPI-MANA), National Institute for Materials Science, Ibaraki, Japan; e Research and Services Division of Materials Data and Integrated System, National Institute for Materials Science, Ibaraki, Japan

**Keywords:** Surface oxidation, GaN, density functional molecular dynamic calculation, MOS structure

## Abstract

Understanding the process of oxidation on the surface of GaN is important for improving metal-oxide-semiconductor (MOS) devices. Real-time X-ray photoelectron spectroscopy was used to observe the dynamic adsorption behavior of GaN surfaces upon irradiation of H_2_O, O_2_, N_2_O, and NO gases. It was found that H_2_O vapor has the highest reactivity on the surface despite its lower oxidation power. The adsorption behavior of H_2_O was explained by the density functional molecular dynamic calculation including the spin state of the surfaces. Two types of adsorbed H_2_O molecules were present on the (0001) (*+c*) surface: non-dissociatively adsorbed H_2_O (physisorption), and dissociatively adsorbed H_2_O (chemisorption) molecules that were dissociated with OH and H adsorbed on Ga atoms. H_2_O molecules attacked the back side of three-fold Ga atoms on the (0001̅) (−*c*) GaN surface, and the bond length between the Ga and N was broken. The chemisorption on the (101̅0) *m*-plane of GaN, which is the channel of a trench-type GaN MOS power transistor, was dominant, and a stable Ga-O bond was formed due to the elongated bond length of Ga on the surface. In the atomic layer deposition process of the Al_2_O_3_ layer using H_2_O vapor, the reactions caused at the interface were more remarkable for *p*-GaN. If unintentional oxidation can be resulted in the generation of the defects at the MOS interface, these results suggest that oxidant gases other than H_2_O and O_2_ should be used to avoid uncontrollable oxidation on GaN surfaces.

## GRAPHICAL ABSTRACT

**Figure:**
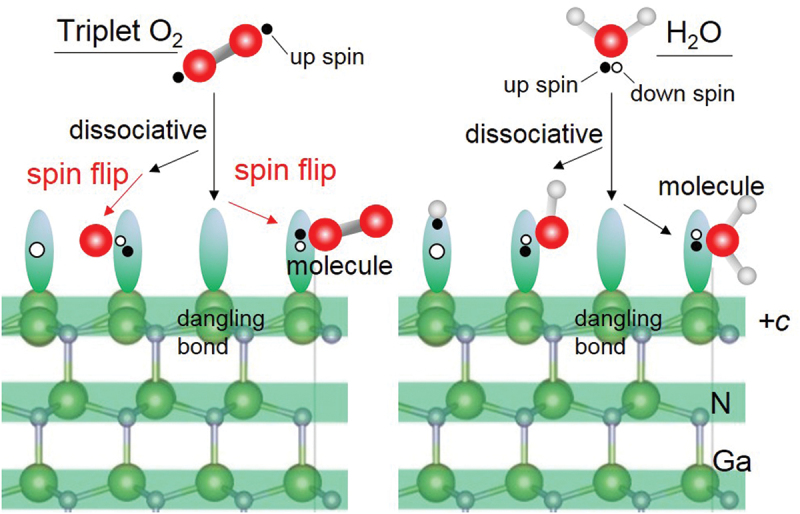

## 1. Introduction

Analyzing the initial stage of oxygen adsorption on GaN surfaces is important in order to precisely control the oxidation and construct fine metal-oxide-semiconductor (MOS) structures of GaN power electronic devices. Conventionally, the vertical type of GaN power MOS transistor has a trench structure [1], because the technique of implanting Mg ions into GaN for regional *p*-type conduction is not yet sufficiently mature. Since GaN film with a smooth surface is conventionally grown along the Ga-face (0001) (*+c*) polar direction, the electron channel is formed on the (101̅0)(*m*) plane of the trench side wall.

To form the MOS structure, a thin layer of an oxide such as SiO_2_ or Al_2_O_3_ on GaN is often deposited by atomic layer deposition (ALD) while introducing H_2_O vapor alternatively as an oxidation gas into the reactor. The defect sites at the interface of Al_2_O_3_/GaN were decreased when O_3_ gas instead of H_2_O vapor was used during ALD [2]. N_2_O plasma treatment may be used to reduce the interface state between Al_2_O_3_ and GaN [3]. Thus, the various crystalline planes of the GaN surface are simultaneously exposed to various oxidant gases during device processing. Since the incorporation of oxygen impurities depends on the crystalline planes of GaN[4], it is important to understand the oxidation process for various surfaces of GaN in order to fabricate a favorable interface between GaN and the oxide layer and improve the performance of the MOS structure.

We have studied the variation of chemically oxidized state on the polar and *m*-plane surfaces of GaN by X-ray photoelectron spectroscopy (XPS) operated under synchrotron radiation to detect O 1s spectra continuously during O_2_ molecule beam irradiation [5]. The dependence of the chemically oxidized state on the GaN surface was explained well by density functional molecular dynamics (DF-MD) calculation taking into consideration the polarity and lone pair electrons on the GaN surfaces.

In this study, we investigated the oxidation of GaN surfaces by the other oxidant gases of H_2_O, N_2_O, and NO by using the same XPS system. Oxygen adsorption on +*c*, N-face (0001̅) (−*c*) and *m*-GaN surfaces caused by H_2_O was simulated by DF-MD calculation using a theoretical model including both electronic spins on the surfaces and the polarity of GaN. It was found that more oxygen was adsorbed on GaN surfaces under irradiation when using H_2_O vapor than when using the other gases. An Al_2_O_3_ layer with thickness of 1 nm was deposited on *p-* and intrinsic (*i*)-GaN by ALD using H_2_O vapor. The influence of H_2_O vapor will be discussed with respect to variation of the valence band structure.

## 2. Experiments and computational details

The samples were +*c*, −*c*, and *m*-GaN bulks with polished surface grown by halide vapor phase epitaxy (HVPE), and +*c*- and *m*-GaN films grown by metalorganic chemical vapor deposition (MOCVD). XPS measurements were carried out at BL23SU, SPring-8 [6]. High-purity O_2_, N_2_O and NO gases diluted with 1% in helium gas were supplied through a nozzle head (φ100 μm) heated at approximately 1400 K. These gases were so pure that no impurity was detected by quadrupole mass spectrometer (QMS). We measured the gas concentration ratio of diluted oxidation gas by QMS, and the flux number listed in Table 1 was estimated according to the previous report [7]. This oxidation condition using O_2_ gas was mild enough to oxidize two monolayers of a Si surface in two hours of irradiation [8]. Since the molecular beam was not applied to H_2_O vapor, it was introduced at room temperature to keep the pressure in the XPS chamber at the same level of ~1 × 10^−5^ Pa corresponding to the same flux of the case of O_2_ gas. The O 1 s core spectrum was continuously detected with one scan for about 30 s at a binding energy of 538 to 525 eV until the end of irradiation. The details of the experiment were described elsewhere [5].

**Table 1.** List of irradiation conditions of oxidant gas during XPS measurement

Oxidant	Pressure (Pa)	Oxidant Flux/He (cm^−2^ s^−1^)	Beam energy (eV)
H_2_O	9.0 × 10^−6^	-	-
O_2_	1.0 × 10^−5^	2.8 × 10^14^/ 9.7 × 10^14^	2.26
N_2_O	1.1 × 10^−5^	1.1 × 10^15^/ 2.2 × 10^15^	2.85
NO	1.8 × 10^−5^	4.0 × 10^14^/ 2.1 × 10^15^	2.12

To clarify the atomistic configuration on GaN surfaces, we performed DF-MD for the H_2_O molecules on the surfaces in the similar way to the O_2_/GaN system [5]. Density functional theory calculation implemented in CP2K [9] was used for all computations in this research. We used the PBE functional [10] with the local spin density approximation (LSD). The hybrid Gaussian (MOLOPT DZVP) and plane wave (500 Ry for cutoff energy) basis set [11], where the valence pseudo-wavefunctions are expanded in Gaussian-type orbitals and the density is represented in a plane wave auxiliary basis, were used with the Goedecker, Teter, and Hutter (GTH) pseudopotentials [12] constructed for the PBE functional. The total energies were calculated at the *Γ* point in a super-cell approach. For DF-MD, canonical sampling through a velocity rescaling (CSVR) thermostat [13] was used with a temperature of 500 K, the same as the experiment for the NVT ensemble, and the time step was set to 0.5 fs. We performed the DF-MD computation for over 80 ps for energy relaxation, followed by 20 ps for the sampling of each system.

Using the computed cell parameters that were validated in our previous report [5], we prepared the *+c/*−*c* slabs (160 atoms) and *m* slab (128 atoms) of GaN with the vacuum region of over 20 Å in the direction perpendicular to each slab. For H_2_O adsorption on the surfaces, we placed 18 H_2_O molecules into the vacuum region between the slabs. All initial structures for simulating H_2_O molecule adsorption on GaN surfaces were prepared by annealing target systems through high-temperature DF-MD (800 K) with the same settings mentioned above for 1.0 ps to accelerate reaching an equilibrium condition.

## 3. Results and discussion

### 3.1 Real time XPS measurement under oxidation gas

**Figure 1.**
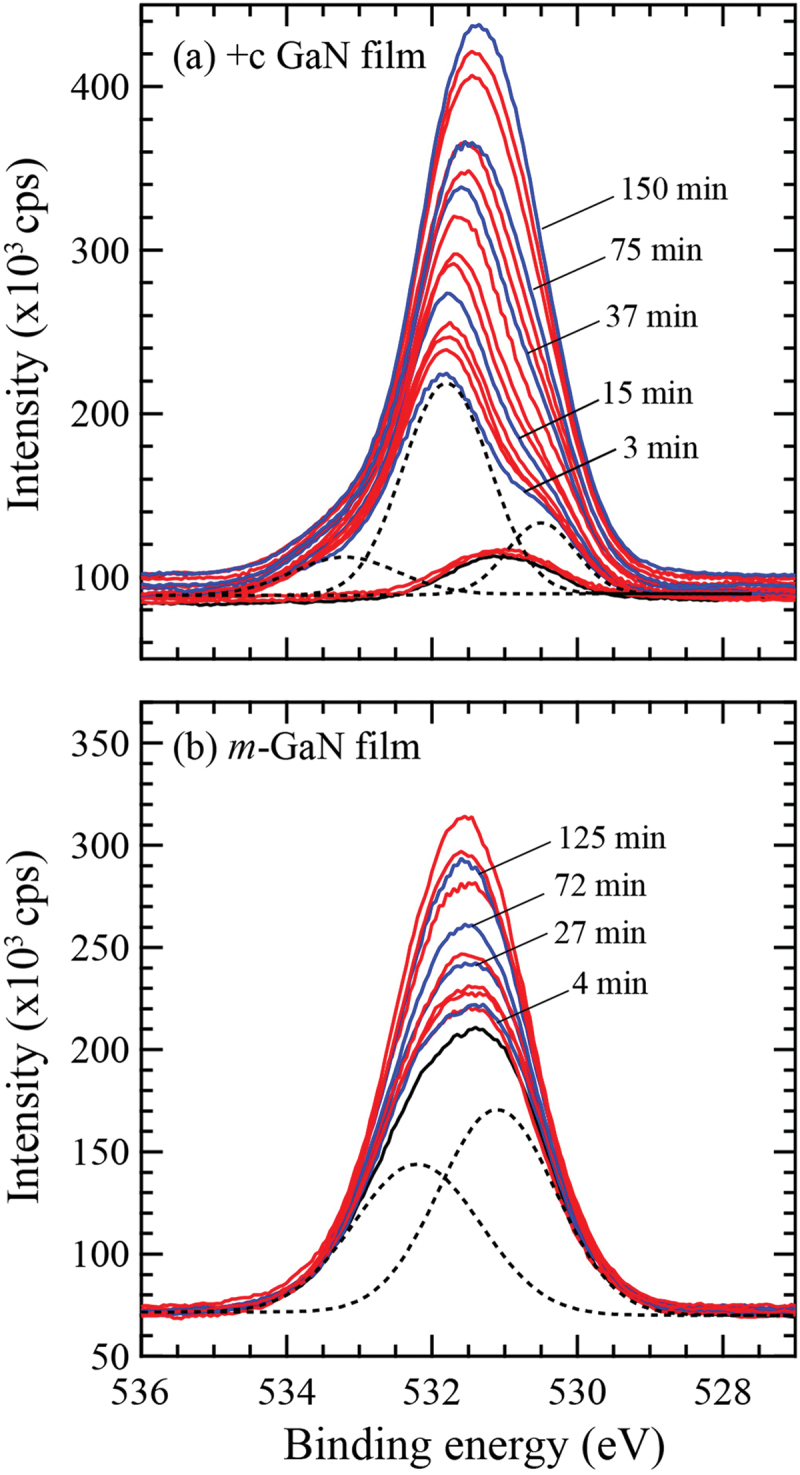
O 1s spectra detected every 30s by XPS under H_2_O ambient at 9.0 × 10^−6^ Pa for (a) +*c* GaN and (b) *m*-GaN surface after annealing at 900°C for 20 min. The initial chemical states are indicated by black lines. The blue lines show the spectra detected at the specific exposure time as indicated in the figure. The dotted lines show the spectra decomposed for *+c* GaN irradiated for 3 min and *m*-GaN after annealing.

Figure 1 shows the variation of O 1s core spectrum detected for *+c* and *m-* GaN films exposed in H_2_O vapor ambient. The spectrum at the initial stage of irradiation was decomposed as indicated by the dotted lines. The signal intensity at higher binding energy (~531.8 eV) increased drastically at the initial exposure for the *+c* GaN surface, while that at the lower binding energy (~530.4 eV) dominantly increased in the case of the O_2_ molecule beam irradiation [5]. For *m-*GaN film, the surface oxygen was hardly removed by annealing at 900°C as shown by the black lines in Figure 1(b). Both chemical states of oxygen at 531.1 eV and 532.2 eV gradually increased with the increase of H_2_O exposure time.

**Figure 2.**
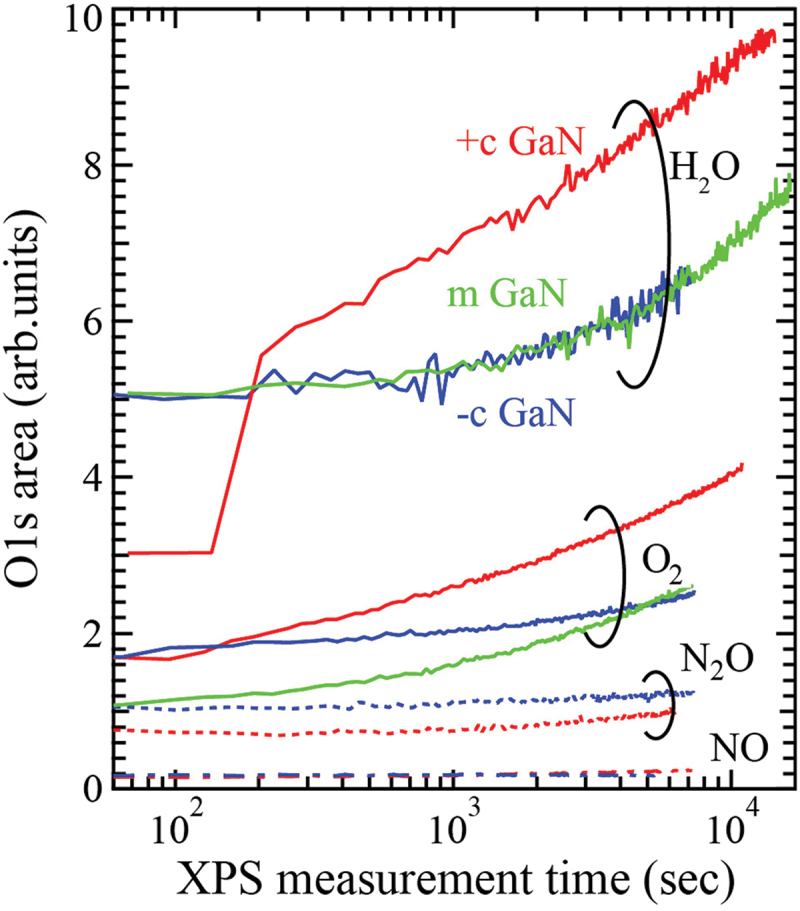
Variation of O 1s area detected by XPS upon exposing *+c* (red), −*c* (blue) and *m-* (green) GaN surfaces to each oxidant gas. The value of the initial area for each gas was shifted to improve legibility. The shutter introducing oxidation gas was open after a few scans of O1s core spectrum in the real-time XPS measurement.

The same experiments using O_2_, N_2_O and NO gas were carried out, and the area of O 1s spectra was estimated. Figure 2 shows the variation of the O 1s area of +*c* (red), −*c* (blue) and *m*- (green) GaN surfaces for each oxidant gas during the measurement time. When N_2_O and NO gases were used, almost no oxygen was adsorbed on the *+c* and −*c* GaN surfaces (so we did not expose them to *m*-GaN). When GaN surfaces were exposed to H_2_O vapor, oxygen was quickly adsorbed on +c GaN surface. The drastic increase at the initial stage is due to the limit of detection because the adsorption speed at the initial stage on *+c* GaN surface was too high to follow the data acquisition correctly. The amount and rate of oxygen adsorbed on the surfaces were larger and higher than in the case of O_2_ gas. The dependence of oxygen adsorption on GaN surfaces was opposite to the oxidation power for bulk metals (H_2_O ≪ O_2_ < NO, N_2_O) under equilibrium condition [14,15]. The DF-MD calculation was carried out in order to understand the larger oxygen adsorption on GaN surfaces caused by H_2_O vapor in spite of its lower oxidation power.

### 3.2 DF-MD calculation for the surface oxidation by H_2_O vapor

#### 3.2.1 *H_2_O on* +c/−c *polar GaN surfaces*

**Figure 3.**
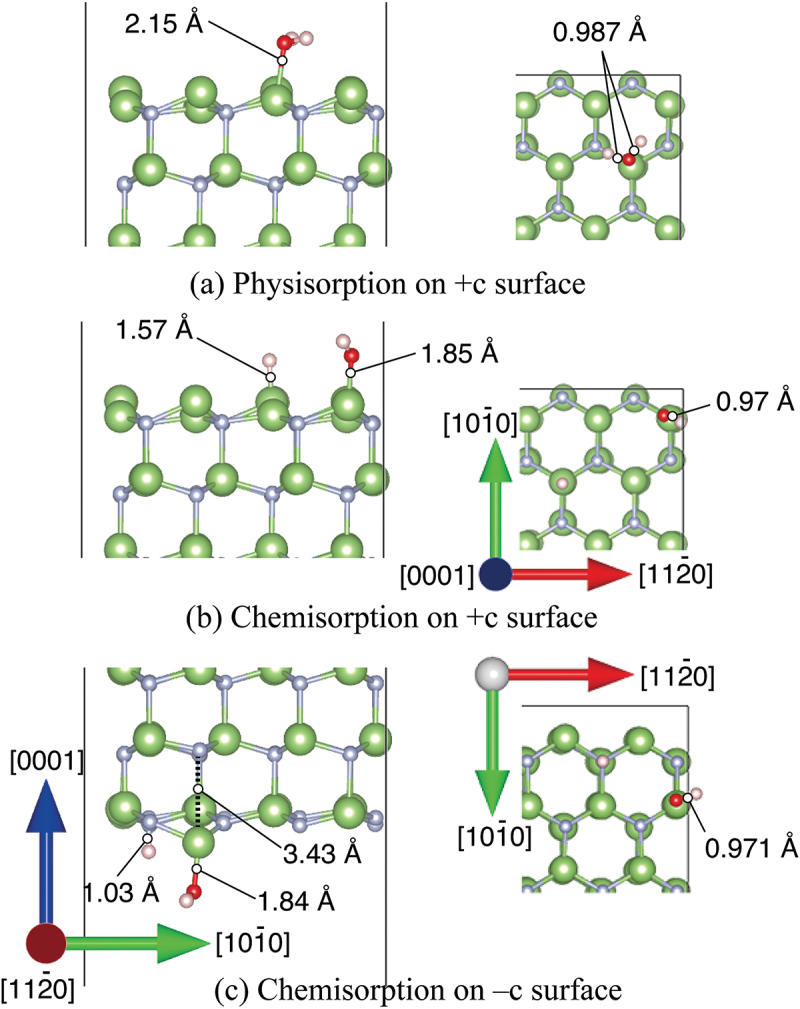
Optimized structures of H_2_O molecule obtained from DFT for (a) physisorption H_2_O and (b) chemisorption H_2_O on *+c* GaN surface, and (c) *−c* GaN surface. Green and gray spheres indicate Ga and N atoms, respectively. Red and pink spheres indicate O and H atoms, respectively. The bond lengths involved in H_2_O adsorption are shown.

Adsorption between H_2_O molecules and GaN surfaces in the intermediate spin state was simulated by DF-MD. On the basis of the structures obtained from DF-MD, we performed structure optimization to obtain the stabilization energy of adsorption on each GaN surface. We found two types of adsorbed H_2_O molecules on the +*c* surface: non-dissociatively adsorbed H_2_O (physisorption), and dissociatively adsorbed H_2_O molecule (chemisorption) that is dissociated to OH and H adsorbed on Ga atoms as shown in Figure 3(a,b), respectively. On the +*c* GaN surface, the O 1s spectra (Figure 1(a)) are consistent with the simulation result that the oxygen dominantly exists in two states, that is, two possible adsorption structures, the dissociation, and adsorption of H_2_O molecules, co-exist. The adsorption energies are listed in Table 2. The chemisorption (2.22 eV) was more stable than physisorption (0.709 eV). Obtained adsorption energy in the *+c* GaN surface was comparable with those in the other report [16] where hydrogen termination was applied to the opposite surface.

**Table 2.** Adsorption energy of H_2_O on GaN surfaces. The corresponding figure number is added after each adsorption energy

Surface	Desorbed (eV)	Chemisorption(eV)		Physisorption (eV)
*+c*	0.0	2.22 (Figure 3(b))		0.709 (Figure 3(a))
–*c*	0.0	1.25 (Figure 3(c))		-
	Desorbed (eV)	Chemisorption 1 (eV)	Chemisorption 2 (eV)	Enhanced surface (eV)
*m*	0.0	6.68 (Figure 5(a))	6.05 (Figure 5(b))	9.69 (Figure 8(b) bottom)

**Figure 4.**
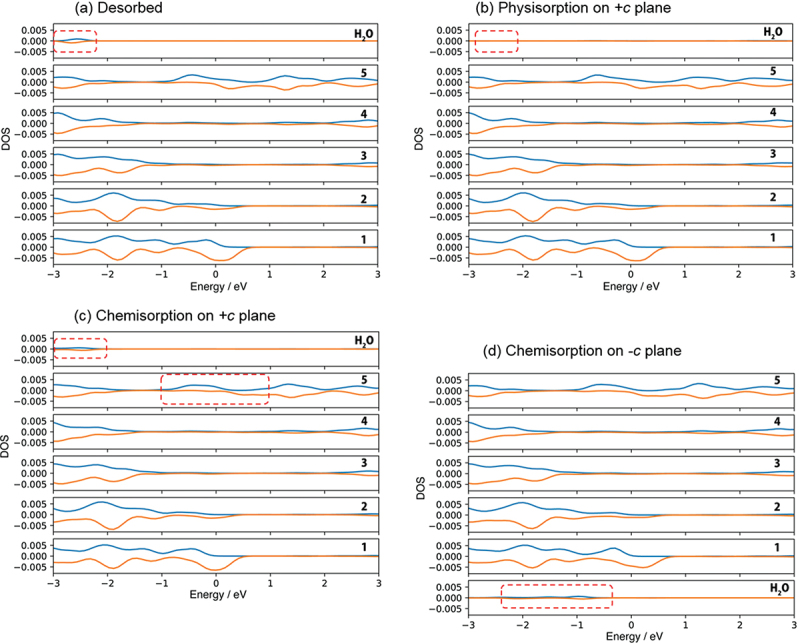
Density of state for (a) no adsorption of H_2_O on *+c**/−**c* GaN surface called ‘Desorbed’, (b) physisorption and (c) chemisorption of H_2_O on *+c* GaN, and (d) chemisorption of H_2_O on – *c* GaN surface. The positive and negative value of DOS indicate alpha and beta electrons respectively. See Fig. 1S-1 for indices of each layer of the GaN slab.

According to the density of state (DOS) analysis, physisorption originates from the donation of the lone pair electrons on H_2_O molecule (The peak of H_2_O at around −2.5 eV in Figure 4(a)) to a Ga ion on the surface (the peak disappears as shown in Figure 4(b)). On the other hand, chemisorption is due to the breaking of the O-H bond of H_2_O molecule to OH radical (DOS of H_2_O in the range from −2.5 to −3.0 eV in Figure 4(c)) and hydrogen radical adsorbed on Ga atom and Ga ion, respectively (DOS of 5^th^ layer in the range from −1.0 to 1.0 eV in Figure 4(c)). Since the state of oxygen at higher binding energy increased faster as shown in Figure 1(a), it is considered that chemisorption is dominant on the *+c* GaN surface.

The DF-MD calculation showed that H_2_O molecules also attack the back side of three-fold Ga atoms on the −*c* GaN surface bonding tetrahedrally with three N atoms. Only dissociative adsorption of OH took place. The dissociated hydrogen is bonded with nitrogen. For the structural optimization, the opposite bond length between N and Ga where dissociative OH adsorbed was expanded to 3.43 Å, indicating bond breaking (Figure 3(c)). This supports the mechanism of selective etching of the −*c* GaN surface in alkali solution as we reported previously [17]. Interestingly, this bond breaking does not largely influence the DOS of the sublayer as shown in layer 2 in Figure 4(d), but the subtle increase of alpha and beta electron density of layer 1 due to the adsorption of OH^−^ (DOS of H_2_O in Figure 4(d)).

#### 3.2.2 *H_2_O on* m*-GaN surface*

**Figure 5.**
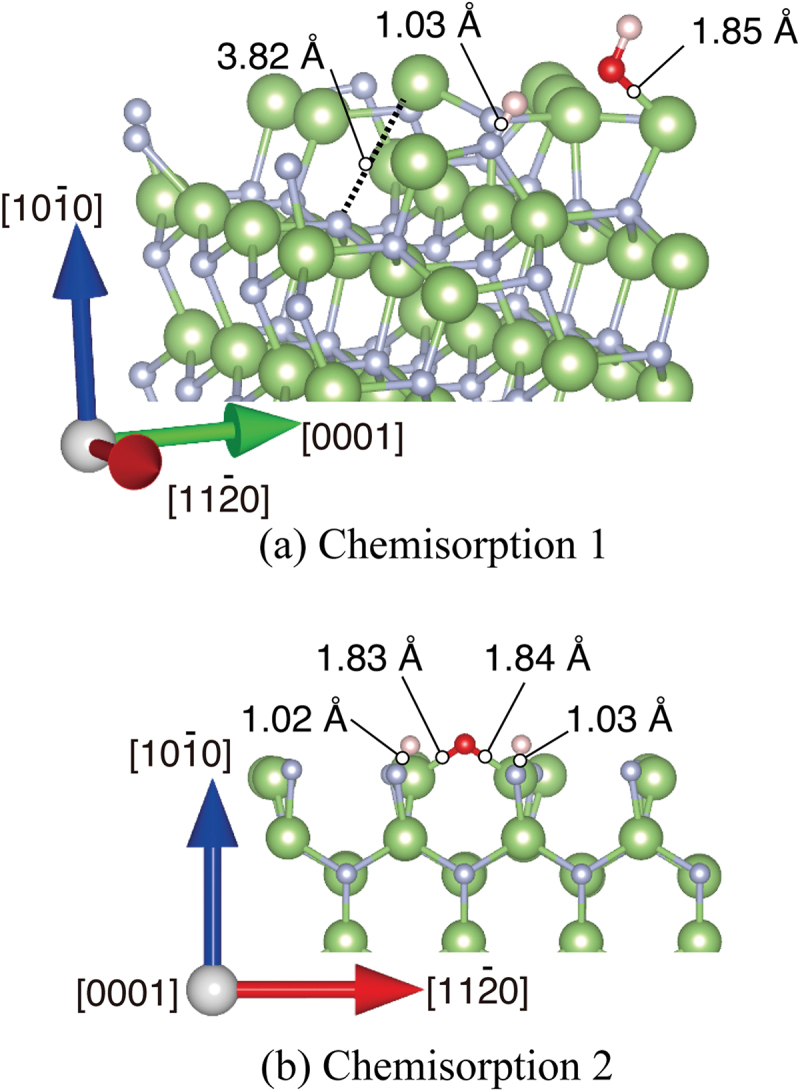
Optimized structures of H_2_O molecule obtained from DFT on *m*-GaN surface exhibiting two types: (a) chemisorption 1 in which reconstruction takes place, and (b) chemisorption 2 forming the GaO bond directly. Green and gray spheres indicate Ga and N atoms, respectively. Red and pink spheres indicate O and H atoms, respectively. The bond lengths and distance relevant to H_2_O adsorption are shown.

The chemisorption of H_2_O also occurs intensively on the *m*-plane of the GaN surface. Ga cation/Ga radical and N cation/N radical coexist on *m*-GaN surface in our model. Therefore, radical electrons rather than dangling bond exist on the Ga sites. Surely, this Ga site shows high reactivity as reflected in the H_2_O adsorption energy shown in Table 2. There are two possible dissociatively adsorbed states. One is the state in which the neighboring Ga loses an electron and one bond with nitrogen in sublayer is broken, as shown in Figure 5(a) (chemisorption 1).

**Figure 6.**
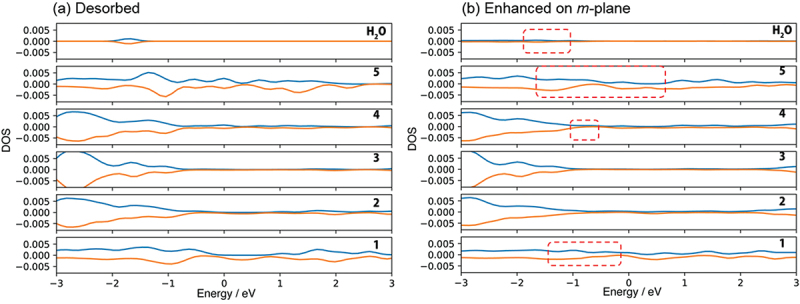
DOS of (a) no adsorption of H_2_O on *m*-GaN surface called ‘Desorbed’, and (b) dissociative H_2_O adsorption on the enhanced *m*-plane of Chemisorption 1. The number on right hand side indicates the layers of *m*-GaN slab model of MD-calculation (See Fig. 1 S-1, Supporting information). The positive and negative value of DOS indicate alpha and beta electrons respectively. See Fig. 1 S-1 for indices of each layer of the GaN slab.

The chemisorption 1 (6.68 eV of adsorption energy) induces the enhanced adsorption site in which another H_2_O molecule attacks the Ga atom on the *m*-GaN surface. The Ga atom near the site of OH adsorption was shifted upward, and one distance between Ga and N atom that lies at the sublayer was 3.82 Å. The Ga atom has higher reactivity. The adsorption energy of H_2_O on this enhanced *m*-plane (9.69 eV) is the largest in the obtained structure shown in Table 2. H_2_O adsorbed quickly on the site of chemisorption 1 due to the Ga atom shifted upward. This enhanced large variation of DOS related to Ga and N atoms as shown with the dotted squares in Figure 6.

The other states are the formation of a Ga-O-Ga bridge bond in the [0001] direction, and two hydrogen atoms adsorbed on nitrogen atoms near the Ga atom in Figure 5(b) (chemisorption 2). The estimated adsorption energy of chemisorption 1 was 6.68 eV, which was more stable than that of chemisorption 2 as listed in Table 2. According to the MD calculation, however, chemisorption 2 was dominant. This fact indicates that the formation of the Ga-O-Ga bridge bond is kinetically favorable on *m*-GaN in actual oxidation by H_2_O vapor.

**Figure 7.**
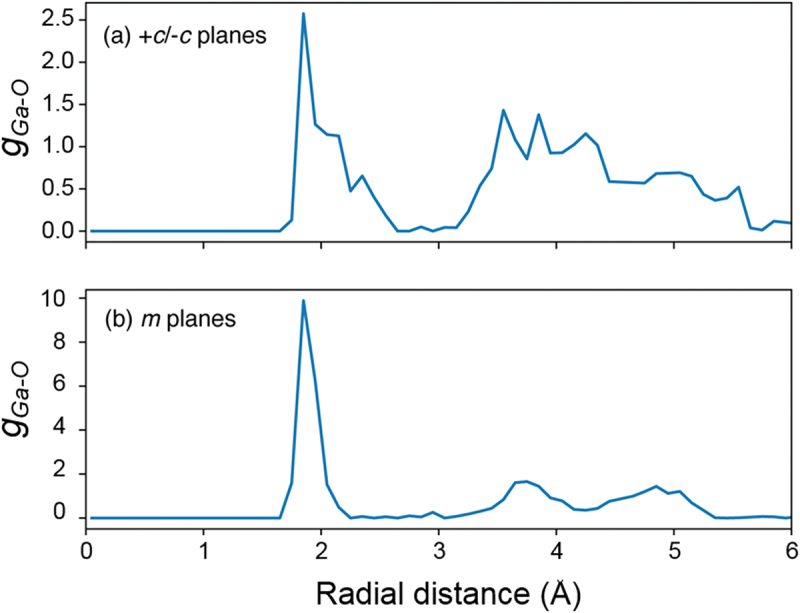
Radial distribution function of Ga-O on (a) *+c**/−**c* planes and (b) *m*-plane using the trajectory obtained by MD calculation during last 90 ps.

Figure 7 shows the radial distribution function (RDF) of Ga-O on *+c**/−**c* planes (a) and *m*-plane (b) using the trajectory during last 90 psec. The RDF of Ga-O on *+c**/−**c*-GaN surface in Figure 7(a) shows broad peaks except for the first peak at around 1.85 Å that means the lower periodicity of Ga-O. In contrast to the case of *+c**/−**c* planes, the RDF of Ga-O on *m*-GaN surface in Figure 7(b) indicates the presence of clear peaks. The first peak appears at around 1.85 Å. The second peak attributes to the oxygen atom of water molecules that adsorbed on Ga-O via hydrogen bond and the third peak appears at around 5.05 Å, which is approximately three times the position of the first peak due to the next neighbor oxygen atom of Ga-O. Hence, the RDF on *m*-GaN surface indicates the periodic structure of Ga-O-Ga. Furthermore, this Ga-O-Ga bride bonds are preserved for 90 ps at 500 K. Therefore, H_2_O molecules adsorbed on the *m*-GaN surface are more stable than the others. This is consistent with the difficulty of removing the oxygen adsorbed on the *m-*GaN surface by thermal annealing.

#### 3.2.3 Spin interaction on GaN surfaces

From the electronic structure, H_2_O molecules can adsorb onto the *+c*/−*c* and *m* surface without spin flip, because H_2_O can choose the spin state of electrons to donate to the surface in contrast to the case of GaN/O_2_ [5]. Thus, the spin interaction between the GaN surface and molecule is important to the oxygen adsorption. Lone pairs of oxygen in H_2_O molecules approach the unoccupied *sp^3^* hybrid orbital of the Ga atom on the +c GaN surface. In the chemisorption, the OH^−^ group can donate one electron with spin opposite to the spin of the dangling bond on the surface without flipping. Consequently, the oxidation was enhanced and its rate was high. On the other hand, since O_2_ was a triplet, spin flipping was necessary for oxidation of the GaN surface to progress, that is, the oxidation speed was lower than in the case of H_2_O. Similar to the O_2_ molecule, the NO molecule has a radical electron, and flipping the spin and/or finding a Ga dangling bond with the opposite spin was needed to bond to the surface. On the other hand, N_2_O has no radical electron but a resonant structure. It is supposed that the energy level of N_2_O must be deeper compared with the Fermi level of GaN surface. This energy level difference may prevent the interaction of electrons between GaN surface and N_2_O.

**Figure 8.**
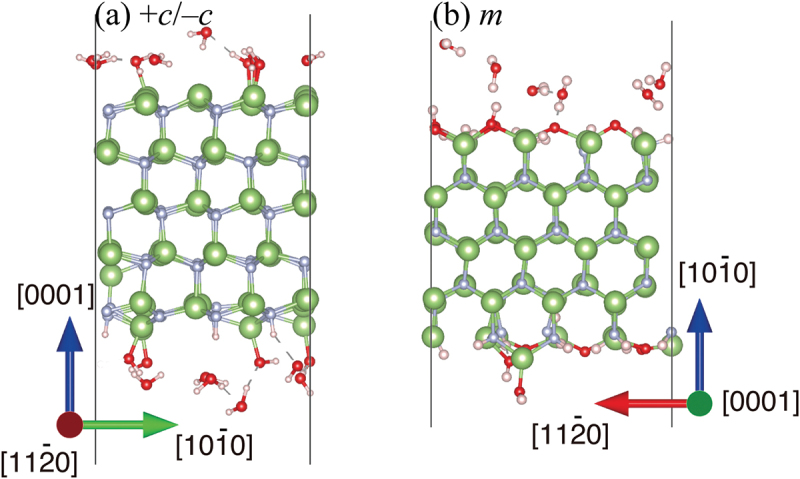
Snapshots of (a) +*c*/−*c* GaN surface slab, and (b) *m*-GaN surface slab interaction with H_2_O molecules during DF-MD at 500 K. Green and gray spheres indicate Ga and N atoms, respectively. Red and pink spheres indicate O and H atoms, respectively.

Figure 8 shows cross-sectional snapshots of the DF-MD simulations of H_2_O molecules interacting on the *+c* and *m*-GaN surfaces. Apparently, large numbers of O, OH, and H_2_O molecules are adsorbed on the GaN surfaces. Assuming a Ga atom as an active site, the coverage (*θ*) on the *+c* surface was 0.4, which was much larger than that in the case of O_2_ molecules (*θ = *0.3) [5]. On the *m*-GaN surface, the coverage was 1.0 and 0.4 for H_2_O and O_2_, respectively. This simulation result was consistent with the experimental result of the larger oxygen adsorption of H_2_O on GaN surfaces in Figure 2.

### 3.3 Influence of Al_2_O_3_ deposited on GaN by ALD using H_2_O vapor

**Figure 9.**
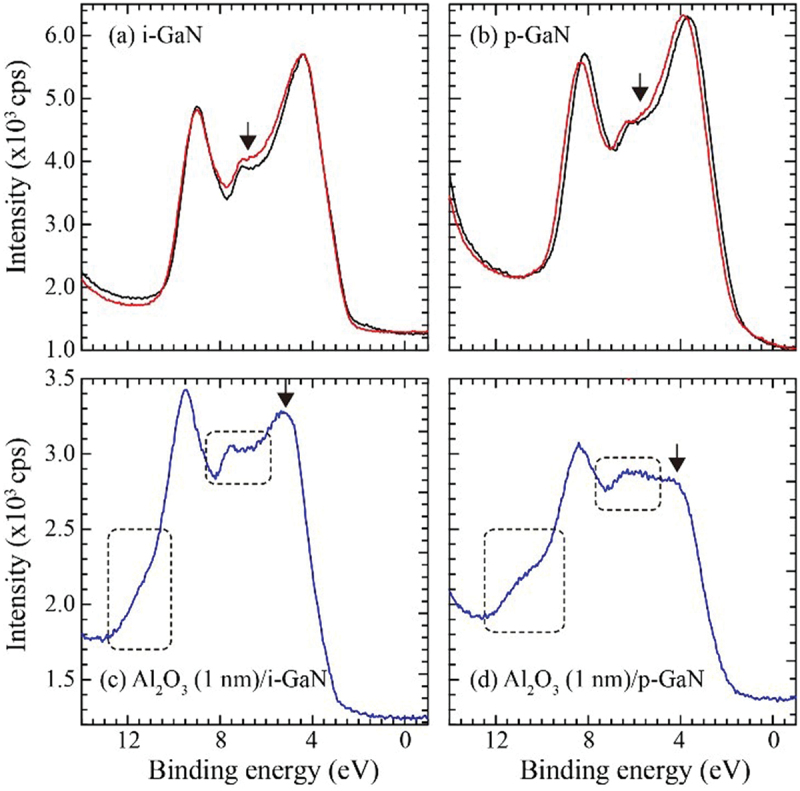
Valence band spectra: (a) *i*-GaN and (b) *p*-GaN exposed to O_2_ molecule beam for 2 hours (red). The black lines represent those of as-installed samples. The intensity at lower binding energy was normalized. (c) Al_2_O_3_ (1 nm)/*i*-GaN and (d) Al_2_O_3_ (1 nm) /*p*-GaN. The Al_2_O_3_ layer was deposited by ALD using H_2_O vapor.

In order to confirm the influence of H_2_O vapor in actual device processing, we deposited an Al_2_O_3_ layer with 1 nm of thickness on MOCVD *i*-GaN and *p*-GaN films simultaneously at 120°C under 10 hPa of pressure by ALD (PICOSUN, SUNALE R-100B, Finland). In this process, water vapor and tri-methyl aluminum (TMA) were introduced in an alternating mode, acted as oxidant and metal precursor, respectively. The pulse and purge time for both precursors were 0.1 and 4.0 s, respectively. The deposition rate was around 0.1 nm/s. Mg-doped GaN films (*+c*) with 1 μm of thickness were grown on c-sapphire substrate at 1040°C by MOCVD (E&E Evolution Co. Ltd., Japan). Amount of Mg was 2 × 10^19^ cm^−3^, and *p*-type carrier density was 1 × 10^18^ cm^−3^. The surface morphology was step and terrace structure (RMS: 7.4 Å). The valence band spectra for the GaN samples were observed by the same XPS system. Figures 9(a,b) show the valence band spectra of *i*-GaN and *p*-GaN without the Al_2_O_3_ layer before and after irradiation of the O_2_ molecule beam for 2 hours. For the valence band, the signals related to oxidation at the energy of around 6 eV [18] as indicated by the arrows were slightly larger after the irradiation. The variation caused by the O_2_ irradiation was not so remarkable, and these valence spectra for the samples were comparable to those of GaN. On the other hand, when the ALD Al_2_O_3_ layer was deposited on the GaN layers, drastic variations in the valence band spectra were observed as shown in Figures 9(c,d). The chemical oxidation states of GaN caused by H_2_O vapor were detected at binding energies in the range of 5–7 eV and 10–12 eV [19] surrounded by the dotted squares in Figure 9. The oxidation was apparently enhanced for *p*-GaN. Actually, *p*-GaN sample with no Al_2_O_3_ layer seemed to have higher reactivity against H_2_O exposure, because the chemical shift caused by oxidation was enhanced as shown in Fig. S2 in Supporting Information. In addition, the intensity of the peak at the binding energy of 4–5 eV as indicated by the arrows in Figure 9 was decreased. We do not understand the origin of this reduction. One possibility is the formation of Al_x_Ga_1-x_N at the interface, because the intensity was reported to be decreased with the increase of Al composition in Al_x_Ga_1-x_N [20]. The interface at the MOS must be confirmed by cross-sectional TEM. It is noticed that this degradation was remarkable at the interface of *p*-GaN.

It is considered that the reactivity of H_2_O vapor on the GaN surface is higher. As reported by Taoka et al [2], the trap states at the interface of Al_2_O_3_ (20 nm) deposited on GaN by O_3_ in ALD were smaller than that by H_2_O vapor. Although O_3_ gas has higher oxidation power, its reactivity on the GaN surface is considered to be lower. This is reason why spin flipping is necessary in a similar way to the case of O_2_ molecules as mentioned in Sec. 3.2.3. There is still controversy as to whether the GaO_x_ layer formed at the interface between the oxide and GaN layers effectively reduces interface defects [20–22]. If unintentional oxidation on GaN surface would influence the generation of interface trap states, use of N_2_O/NO in the process of depositing oxide layer instead of H_2_O is alternative approach, especially for the MOS interface at the *p*-GaN layer. However, since N_2_O/NO gases have much higher oxidation power, it is difficult to control the growth mode and the pre-reaction with TMA in gas phase. The conditions to deposit oxide layer by process using N_2_O/NO should be optimized, as the reported in the case of ZnO film grown by MOCVD using di-ethyl Zn and oxidation gas in H_2_ ambient [15,23] to obtain smooth surface.

## 4. Conclusion

We investigated the behavior of oxidation on GaN surfaces by both real-time XPS and DF-MD simulation. H_2_O vapor has the highest reactivity on GaN surfaces in H_2_O, O_2_, N_2_O and NO gases. This is strongly related to the spin interaction between H_2_O and GaN surfaces. The dissociation and adsorption of H_2_O molecules co-exist on the *+c* surface. The bond length between the Ga and N on the −*c* GaN surface was increased by OH attacking the back side of three-fold Ga atom. The chemisorption on the *m*-plane of GaN was dominant, and a stable Ga-O bond was formed on the surface. Degradation was remarkable at the interface between *p*-GaN and the Al_2_O_3_ layer deposited by ALD using H_2_O vapor. In order to avoid unintentional oxidation during the deposition process of the oxide layer by ALD, it is suggested that an oxidant gas other than H_2_O and O_2_ should be used.

## Supplementary Material

Supplemental Material
