# Supplementary material for: High reactivity of H2O vapor on GaN surfaces
Source: Sci Technol Adv Mater. 2022 Apr 8;23(1):189–98. doi: 10.1080/14686996.2022.2052180 (PMC9004525; doi:10.1080/14686996.2022.2052180)
Supplement: Supplemental Material [file TSTA_A_2052180_SM6718.docx]

Supporting information

***High reactivity of H_2_O vapor on GaN surfaces***

Masatomo Sumiya^1,*^, Masato Sumita^2^, Yasutaka Tsuda^3^, Tetsuya Sakamoto^3^, Liwen Sang^4^, Yoshitomo Harada^5^, and Akitaka Yoshigoe^3^

*^1^ Next Generation Semiconductor Group, National Institute for Materials Science, 1-1 Namiki, Tsukuba 305-0044, Japan*

*^2^ Center for Advanced Intelligence Project (AIP), RIKEN, 1-4-1 Nihonbashi, Chuo-ku, Tokyo 103-0027, Japan*

*^3^ Materials Sciences Research Center, Japan Atomic Energy Agency, 1-1-1 Kouto, Sayo, Hyogo 679-5148, Japan*

*^4^ International Center for Materials Nanoarchitectonics (WPI-MANA), National Institute for Materials Science, 1-1 Namiki, Tsukuba, Ibaraki 305-0044, Japan*

*^5^ Research and Services Division of Materials Data and Integrated System, National Institute for Materials Science, 1-1 Namiki, Tsukuba, Ibaraki 305-0047, Japan*

* Corresponding author. Email: SUMIYA.Masatomo@nims.go.jp

**S1 DOS of H_2_O adsorption on GaN surfaces obtained by MD calculation**

We calculated the DOS for each surface and adsorption states near the Fermi level using the models in Fig. 1S.


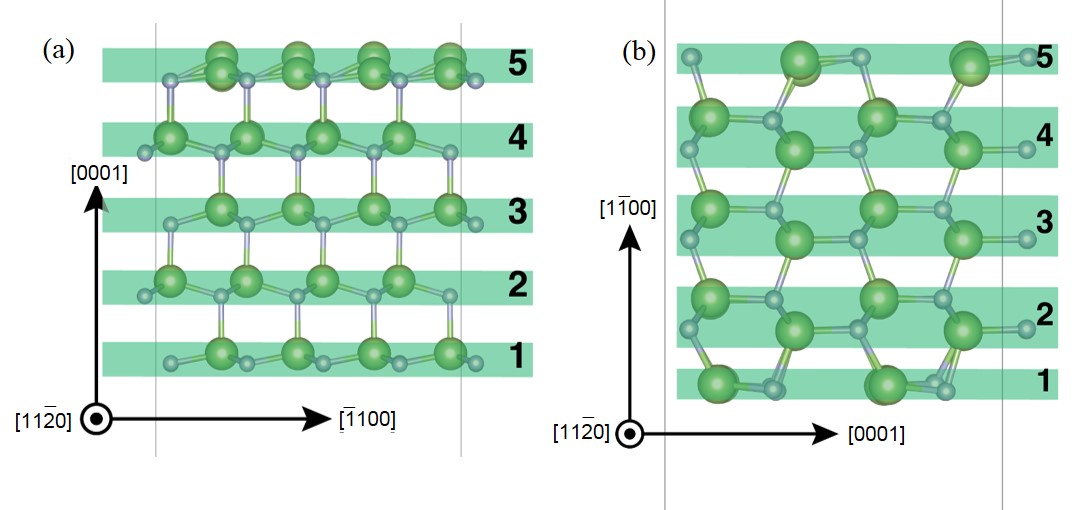


**Fig. 1S-1** Models of GaN surfaces for MD calculation. Large and small spheres show Ga and N atom, respectively. Reproduced by permission from J. Phys. Chem. C 124, 25282 (2020), copyright 2020, American Chemical Society.


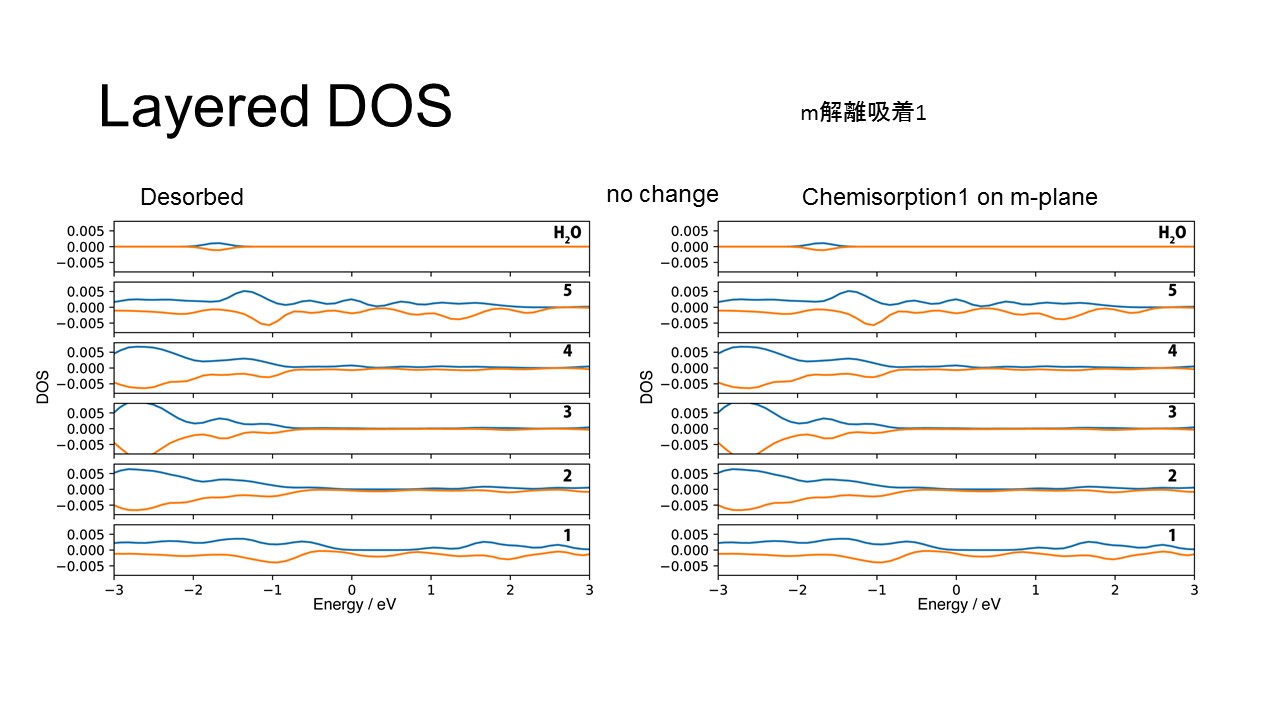


**Fig. 1S-2** DOS of H_2_O chemisorption 1 on *m*-GaN surface.

There is no change of DOS for the chemisorption 1 on *m*-surface. The adsorption took place without exchanging electron between the surface and H_2_O, indicating the high adsorption speed. In this chemisorption 1, Ga atom was shifted upward, breaking the bond. Since the electron was reduced, it is supposed that the chemisorption of H_2_O took place quickly.

**S2. Raw data of core spectra for *p*-GaN and *i*-GaN oxided by H_2_O**


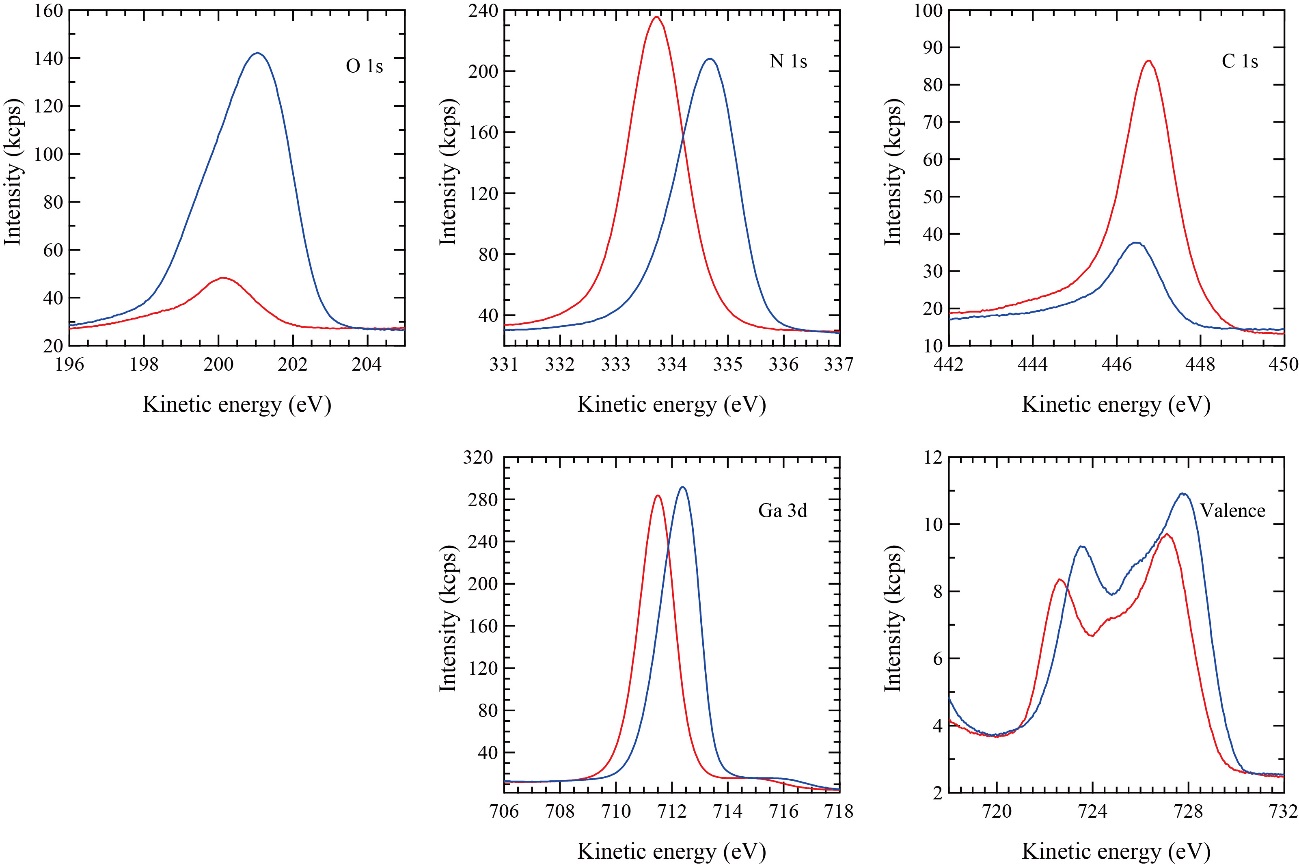


**Fig. 2S.1** Raw data of core spectra (kinetic energy) for *i*-GaN exposed to H_2_O. Red and blue lines indicate XPS data from the sample annealed at 900 °C in XPS chamber in a vacuum and exposed to H2O for 2h 40 min, respectively. X-ray energy was 729.5 eV.


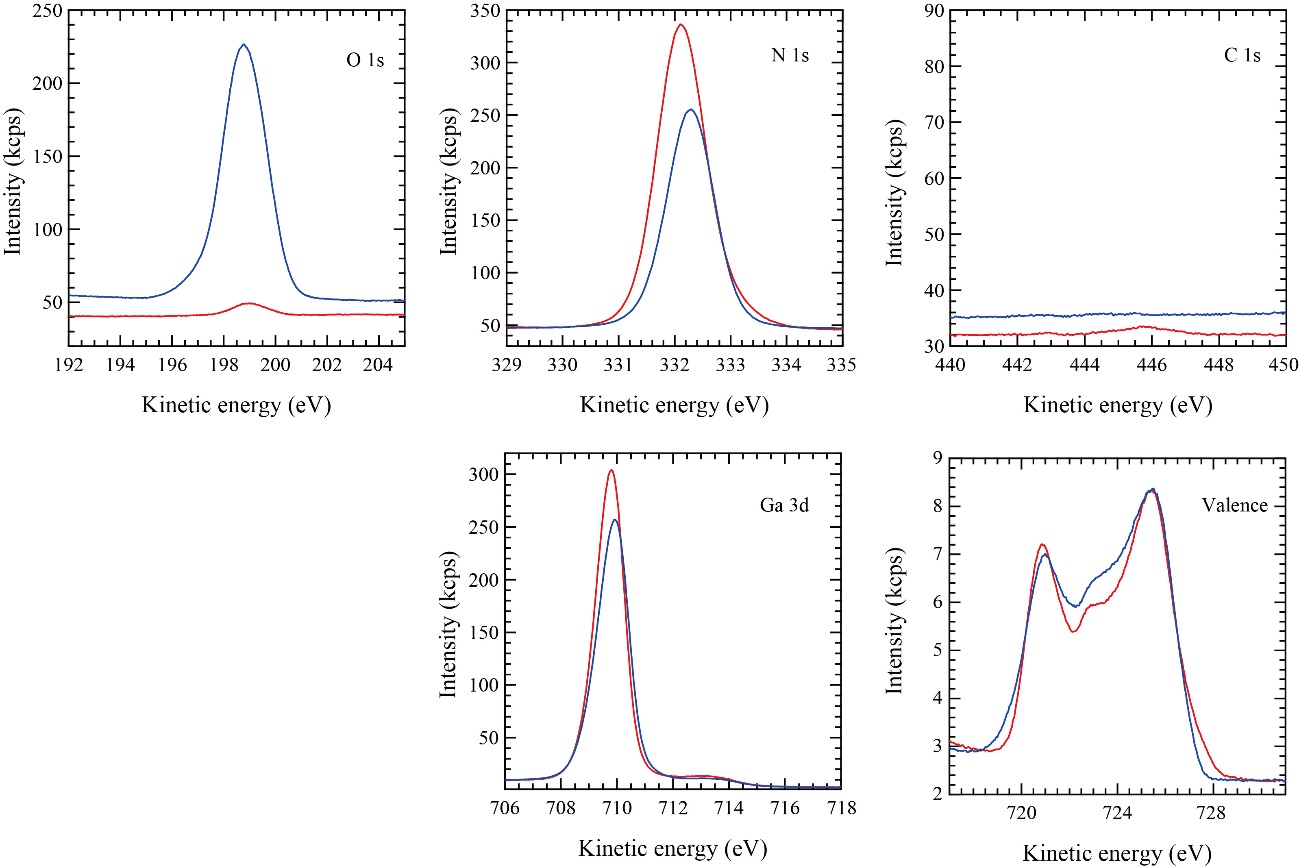


**Fig. 2S.2** Raw data of core spectra (kinetic energy) for *p*-GaN exposed to H_2_O. Red and blue lines indicate XPS data from the sample annealed at 900 °C in XPS chamber in a vacuum and exposed to H_2_O for 2h 6 min, respectively. X-ray energy was 729.5 eV.
